# Brentuximab-induced pneumonitis and organizing pneumonia: a case report with literiture review

**DOI:** 10.1097/MS9.0000000000001878

**Published:** 2024-03-04

**Authors:** Omar R. S. Khalil, Shatha M.A. Mallah, Fahed Owda, Hamza Salim, Haneen Mallah, Jehad Azar

**Affiliations:** a Faculty of Medicine, Al-Quds University, Jerusalem; b Faculty of Medicine, An-Najah National University, Nablus, Palestine; c University of Florida, Gainesville, FL; d Mayo Clinic Hospital, Scottsdale, AZ

**Keywords:** brentuximab vedotin, interstitial pneumonitis, organizing pneumonia

## Abstract

**Introduction and importance:**

Brentuximab vedotin (BV) is an anti-CD30 antibody approved for various cancers, including refractory Hodgkin lymphoma (HL), anaplastic large-cell lymphoma (ALCL) among others. In general, BV has been found to be well-tolerated, with the most frequently reported side effects being peripheral neuropathy and neutropenia. BV-induced pneumonitis is extremely rare. To the best of our knowledge, this is the sixth reported instance of BV-induced lung toxicity.

**Case presentation:**

This case presents a female patient in her forties diagnosed with cutaneous T-cell lymphoma undergoing BV treatment. She developed acute hypoxic respiratory failure, ultimately, underwent a diagnostic evaluation including a computed tomography (CT) scan, which showed bilateral airspace consolidations and ground-glass opacities, suggestive of organizing pneumonia and diffuse alveolar damage. Bronchoscopy with bronchoalveolar lavage and transbronchial biopsy ruled out infection, and pulmonary lymphoma and confirmed the diagnosis of BV-induced pneumonitis. The patient had significant clinical improvement after stopping the offending agent, and starting steroids, with optimal clinical recovery at 8 weeks follow-up.

**Clinical discussion:**

Drug-related pneumonitis poses a significant concern in the management of cancer patients. Numerous chemotherapeutic agents, such as bleomycin, cyclophosphamide, methotrexate, thalidomide, and others, have been associated with pulmonary-related toxicities. These adverse effects primarily stem from direct toxicity or immunosuppression-related infections. Less commonly, immune-mediated injury may occur.

**Conclusion:**

Physicians must have a high index of suspicion for BV-induced pneumonitis, hence, early recognition with subsequent holding of the causative agent, initiation of immunosuppression with steroids, and occasionally steroid-sparing medications, prevent an otherwise fatal outcome.

## Introduction

Highlights

- Brentuximab vedotin is an anti-CD30 antibody approved for various cancers, It is generally well-tolerated and most reported side effects include neutropenia and peripheral neuropathy.
- Lung toxicity is frequently reported as a possible side effect of many chemotheraputic agents. It may present as acute or chronic infections due to immunosuppression or less commonly as an immune-mediated injury which can also contribute to lung-related complications. Rarely, it may present as the form of cryptogenic organizing pneumonia.
- Among the commonly reported agents as the cause of chemotherapy-induced lung toxicity, brentuximab is considered a relatively uncommon cause with only few case reports have reported this instance. To best of our knowledge, our case is the sixth reported case worldwide.
- According to the severity of the condition, the theraputic approach should include temporary or permanent discontinuation of the offending agent, high-dose steroids which may be combined with steroid-sparing agents like Cellcept. Refractory cases may be treated with infliximab and Tocilizumab.

Brentuximab vedotin (BV), an anti-CD30 antibody conjugated with a protease-cleavable linker to monomethyl auristatin E, a microtubule disrupting agent, has garnered approval for treating refractory or relapsed Hodgkin lymphoma (HL) following autologous stem cell transplantation. Additionally, BV has emerged as a significant therapeutic option for anaplastic large-cell lymphoma (ALCL). Ongoing research suggests potential applications of BV in the management of refractory or relapsed cutaneous T-cell lymphomas, diffuse large B-cell lymphomas, and other related conditions^1,2^.

Organizing pneumonia (OP) is a type of interstitial pneumonia characterized by an acute or subacute clinical progression and a histological pattern consistent with acute lung injury^3^. When OP occurs as a result of a clearly identifiable cause, typically following a recent infection of the peripheral bronchial system, it is referred to as secondary OP. Conversely, when no specific cause can be identified as directly responsible for the condition, it is termed cryptogenic OP^3,4^. It typically manifests with a characteristic clinical presentation that includes symptoms such as a dry cough, fatigue, fever, shortness of breath, and unintentional weight loss. A mild flu-like illness often precedes these symptoms. Along with clinical presentation, the diagnosis of OP typically relies on the evaluation of radiological and histopathological findings. Typically, the radiological appearance of OP manifests as widespread airspace involvement with patchy consolidation and ground-glass opacities primarily concentrated in the lower lung zones. These bilateral and peripheral findings tend to exhibit migratory patterns. Among the observed radiological features, the atoll sign, colloquially referred to as the “reversed halo sign,” is commonly identified in cases of OP^4,5^. The transbronchial biopsy can also reveal diagnostic histopathological findings, including excessive fibrotic tissue deposition in alveolar sacs, alveolar ducts, and bronchioles, along with intraluminal Masson bodies^4,6^. Corticosteroids serve as the primary treatment approach and are associated with a favourable prognosis, particularly when the condition is promptly diagnosed^4^.

The lungs are the most frequently involved organs in chemotherapy-related complications, primarily due to either drug toxicity or, more commonly, infections caused by immunosuppression. Less commonly, immune-mediated injury can also contribute to lung-related complications. Radiological patterns commonly observed in drug toxicity encompass interstitial infiltrates, diffuse alveolar damage (DAD), nonspecific interstitial pneumonia (NSIP), eosinophilic pneumonia, OP, pulmonary haemorrhage, as well as oedema and hypertension. Bleomycin, cyclophosphamide, methotrexate, thalidomide, lenalidomide, pomalidomide, and everolimus are frequently identified as the primary drugs associated with OP^4,7^. However, BV is not among the commonly reported causes, with only a few cases documented in the literature. To the best of our knowledge, this case represents the sixth reported occurrence of BV-induced pneumonitis, and it is the first documented instance of BV-induced OP in the medical literature.

## Case presentation

A female patient in her forties was recently diagnosed with cutaneous T-cell lymphoma (Sezary syndrome) for which she had initiated BV treatment. Approximately 2 weeks after receiving the third dose of BV, she developed a productive cough with expectoration of blood-tinged sputum in moderate amounts and increased shortness of breath. Initially, her dyspnoea was exertional but progressively worsened over the course of a few days, leading to severe dyspnoea at rest. As a result, she sought urgent medical attention in the emergency department (ED). Upon admission to the ED, the patient exhibited respiratory distress, and her oxygen saturation dropped to 80% on room air as measured by pulse oximetry. Additionally, she displayed working accessory muscles of respiration and was tachypneic with an elevated respiratory rate of 30 breaths per minute. While her blood pressure remained stable at 130/75, she presented with sinus tachycardia at 125 beats per min. The patient was afebrile, alert, and oriented, with no neurological deficits noted during the examination. Physical examination revealed bilateral decreased air entry, mid-lung zone bronchial breathing, and fine crepitations. Auscultation of her heart detected regular rapid heart sounds, with no murmurs or additional sounds. Skin examination revealed diffuse skin lesions with hyper-pigmentation consistent with cutaneous T-cell lymphoma, while the rest of her physical exam appeared unremarkable.

Subsequently, the patient was admitted to the medical ICU (MICU) due to acute hypoxic respiratory failure, necessitating the use of a high-flow nasal cannula at 60% of the fraction of inspired oxygen (FIO_2_) with a flow rate of 35 liters per min. She denied experiencing any fever, chills, or chest pain but did report exertional palpitations of gradual onset and offset, which were relieved by rest. There was no history of loss of consciousness, abdominal swelling, or lower limb oedema. The patient did not mention any new lumps, swelling, or cutaneous eruptions. Notably, aside from BV, which had been initiated a few months before her admission, there were no recent changes in her medication regimen.

The patient’s previous medical history revealed significant comorbidities, including hypertension, chronic renal failure stage II, and hyperlipidemia. Her psychosocial history is free and she has no family history of lung diseases, connective tissue diseases, or malignancies. Laboratory tests showed evidence of acute prerenal failure, with a creatinine level of 1.27. Creatinine kinase levels were within the normal range, and there was mild leukocytosis at 12 600/µl with no eosinophilia. Additionally, C-reactive protein (CRP) was elevated at 1.6 mg/dl, while the erythrocyte sedimentation rate (ESR) remained within normal limits. Extensive rheumatological evaluation yielded inconclusive results, with negative findings for antinuclear antibodies (ANA), rheumatoid factor, anti-SCL-70 antibodies, anti-SSA, and anti-SSB. Furthermore, the polymyositis and dermatomyositis panel returned negative results.

**Figure 1:**
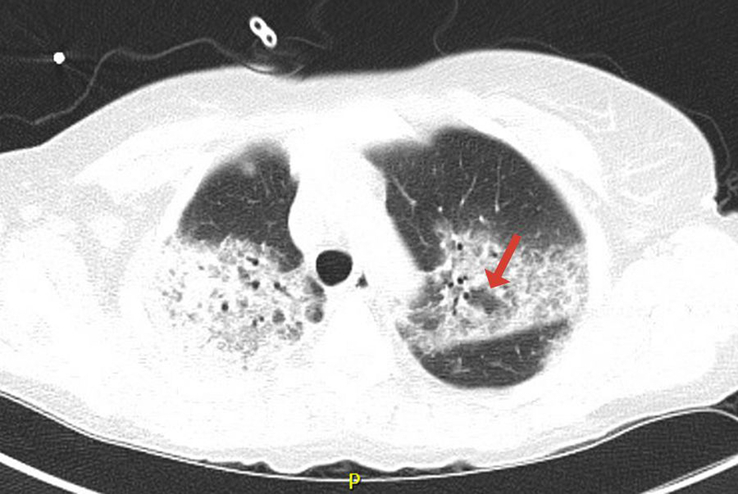
Computed tomography chest shows bilateral upper lobe consolidation, ground-glass opacities, interseptal thickening, and atoll sign (red arrow).

**Figure 2:**
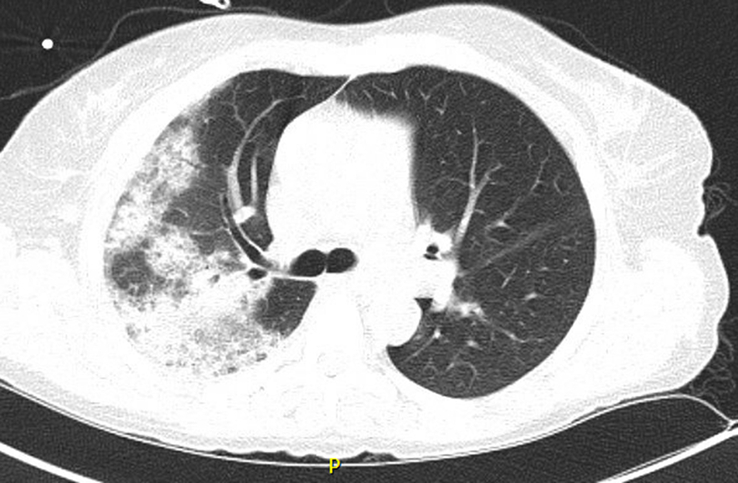
Computed tomography chest shows airspace consolidations involving the right upper lobe, apical and posterior segments, and the superior segment of the right lower lobe.

A comprehensive noninvasive infectious evaluation was conducted, encompassing various tests such as the respiratory viral panel, PCR for SARS-COVID-19, sputum culture, urine legionella antigen, urine histoplasma antigen, and pneumococcal antigen testing, all of which returned negative results. The serology for mycoplasma also yielded negative findings, as did the induced sputum test for pneumocystis jiroveci (PJV) pneumonia, the plasma PCR for CMV, the serum 2,3 D glucan test, and the serum galactomannan test. Additionally, the fungal battery results were negative. In response to the clinical presentation, BV administration was discontinued, and empiric broad-spectrum antibiotics, namely ceftriaxone and azithromycin, were initiated; however, no clinical improvement was observed. Computed tomography (CT) scans of the chest revealed bilateral airspace consolidations, ground-glass opacities, and a reversed halo sign (atoll sign), suggestive of (OP) and DAD (Figs. 1 and 2).

Bedside echocardiography revealed normal left ventricular size and systolic function, with an ejection fraction of 60%, along with normal left ventricular diastolic function and normal right ventricular size and systolic function. No valvular heart disease or signs of pulmonary hypertension were detected, thereby minimizing the possibility of pulmonary venous congestion.

Despite conservative management, the patient showed no clinical improvement, and considering the broad differential diagnosis, which included opportunistic respiratory infections, diffuse alveolar haemorrhage (DAH), and pulmonary lymphoma with lymphangitic spread in the context of Sézary syndrome, BV-induced pneumonitis with DAD and OP were also considered. In light of these considerations, a bronchoscopy with bronchoalveolar lavage (BAL) and transbronchial biopsy (TBBX) from the right upper lobe was performed.

During the bronchoscopy, the airway examination was normal, and no signs of DAH were observed. The BAL sample exhibited 250 nucleated cells, consisting of 69% macrophages and 26% lymphocytes. Bacterial and fungal cultures from the BAL were negative, as were acid-fast bacilli (AFB) tests for tuberculous and non-mycobacterial pathogens. Additionally, PCR tests for cytomegalovirus (CMV) and PJV returned negative results. Galactomannan testing for aspergillus was also negative, as well as the COVID-19 PCR test. Furthermore, the culture of Nocardia showed no growth. The pathology results were consistent with OP with granulation tissue deposition in the distal alveolar space along with intraluminal Masson bodies.

The Grocott methenamine silver (GMS) stain for fungal organisms returned negative results. The histopathological examination of the transbronchial biopsies did not reveal any indications of infection, neoplasms, granulomas, or signs of pulmonary lymphoma. However, the pathological findings were consistent with DAD and OP, confirming the diagnosis of BV-induced pneumonitis with OP and DAD.

Following the diagnosis, the patient’s treatment plan included the initiation of intravenous methylprednisone at a dose of 1 mg per kg twice daily, leading to subsequent clinical improvement. She continued to require high-flow O_2_ supplementtion via nasal cannula for a few days. After 5 days of receiving intravenous methylprednisone, her treatment was switched to oral prednisone at a dosage of 60 mg daily, which was gradually tapered over a 6-week period. During her recovery, she was transferred to a regular nursing floor, where she continued to show marked improvement until the time of discharge. At discharge, the patient was on a prednisone taper regimen and prescribed trimethoprim-sulfamethoxazole prophylaxis.

Upon the 8-week follow-up at the outpatient pulmonary clinics, the patient exhibited clear signs of full recovery. She had returned to her baseline state, with no cough, or shortness of breath. Her functional capacity had improved, and her oxygen saturation measured 96% on room air. Vital signs were stable, and spirometry, lung volume, and diffusion capacity of carbon monoxide were all within normal limits. A follow-up CT scan of the chest, conducted after 6 weeks of systemic steroid treatment, displayed complete radiological resolution, with the lung parenchyma appearing normal.

Following the diagnosis, the patient’s treatment approach involved grade IV BV-induced pneumonitis management. BV was replaced with a different immunomodulator, leading to subsequent remission of her cutaneous T-cell lymphoma, with no signs of pneumonitis relapse observed after discontinuing steroid therapy.

## Discussion

Over the past few decades, the clinical application of innovative anticancer medications has revolutionized cancer therapy, offering remarkable efficacy and efficiency. However, these novel agents are not without their associated adverse effects^8^. Among various toxicities, drug-related pneumonitis (DRP) remains a significant clinical concern in the management of cancer patients^8^. In the present era of precision oncology, the significance of pneumonitis arising from molecular targeted therapy and immune-checkpoint inhibitors (ICI) has garnered acknowledgement in clinical practice^8^. BV is an antibody-drug conjugate (ADC) that selectively targets the CD30 membrane receptor, a member of the tumour necrosis factor receptor superfamily, making it an ideal candidate for ADC-based therapy due to its high expression on specific tumour cells^9^. In clinical trials, the administration of BV was found to be generally well-tolerated, and the most frequently reported adverse events, including peripheral neuropathy and neutropenia, were manageable through dose reductions, only a limited number of nonspecific pulmonary adverse effects were observed^1,9^. However, several reported cases have indicated severe toxicities, such as severe pneumonitis^10–14^.

The pathogenesis of drug-induced lung injury can be attributed to either cytotoxicity or immune-mediated mechanisms^7^. Cytotoxicity may lead to direct damage to the pneumocytes or vascular endothelium, which can be triggered by the generation of reactive oxygen species and cytokines, or by inadequate deactivation of drug metabolites^7^. In cases of DRP, the spectrum of symptom severity can vary from mild manifestations to life-threatening conditions with the potential for rapid progression leading to fatality^15^.

Immunotherapy-related pneumonitis can be categorized into four grades, each presenting variable signs and symptoms, as well as distinct approaches to work-up, management, and follow-up based on the grade^16^. Grade I encompasses patients who are asymptomatic, with lung involvement confined to one lobe or less than 25% of the lung parenchyma^16^. The recommended management entails discontinuing the offending drug, performing a repeat pulmonary function test (PFT) if baseline data is available, offering weekly history and physical examinations (H&P), along with pulse oximetry assessments, and repeating chest CT scan after a four-week interval. Drug resumption is permissible only after full radiological and clinical recovery is confirmed^16^.

For patients classified as Grade II, who exhibit symptoms such as cough, dyspnoea, chest pain, hypoxaemia, fever, or weakness, and whose lung involvement exceeds one lung lobe or constitutes 25–50% of the lung parenchyma, in addition to limitations in instrumental activities of daily living (ADLs), a different management approach is recommended^16^. This includes discontinuing the causative drug, and initiating oral prednisone at a dose of 1–2 mg/kg/day until the patient’s status improves to Grade I or lower, followed by a gradual 4–6-week tapering period. Consideration should be given to empiric antibiotics, pulmonary consultation, and monitoring with H&P and pulse oximetry every 3–7 days. Drug resumption may be considered upon clinical recovery, but in the absence of improvement after 48–72 hours, the condition should be treated as Grade III^16^. Grade III is defined by severe symptoms necessitating hospitalization, consolidations affecting all lung lobes or exceeding 50% of the lung parenchyma, and constraints on self-care ADLs. Patients in Grade III typically receive management involving the permanent cessation of the causative drug, admission to the hospital, and initiation of intravenous methylprednisolone at a dose of 1–2 mg/kg/day, followed by transition to oral prednisone once improvement is observed, with a subsequent 6-week tapering period^16^. Additionally, empiric antibiotics should be considered, and close monitoring with PFTs and consultations with pulmonary and infectious diseases specialists are strongly advised^16^. The causative agent should never be prescribed again, and an alternative medication is recommended^16^. Grade IV is characterized by urgent, life-threatening respiratory compromise that may require intubation, along with findings of lung involvement and limitations akin to Grade III. The management for Grade IV mirrors that of Grade III. However, if no improvement is evident after 48 hours of high-dose steroids, Infliximab at 5 mg/kg once (with the possibility of repetition after 14 days if necessary), Mycophenolate mofetil at 1–1.5 g twice daily, or Tocilizumab at 4 mg/kg should be considered^16^.

In our case, the patient has grade IV immunotherapy-related pneumonitis presenting with acute hypoxemic respiratory failure and requiring MICU management, BV was permanently stopped, the patient was started on IV steroid then switched to oral prednisone as per protocol with clinical improvement to full recovery upon discharge.

Following an extensive review of the available literature (Table 1A, B), we have identified five documented instances of BV-induced lung toxicity^10–14^. Hence, our present case represents the sixth reported occurrence, to the best of our knowledge.

**Table 1:** (A, B) provides a comprehensive summary of the previously reported cases, facilitating a comparative analysis with our own case*

(A)								
Number\ authors	Age\sex	Presentation	Onset	CT findings	Bronchoscopy	Lung biopsy	Has DAH, OP or not	Required O2, ICU?
1\ Sabet *et al*.^10^	29\female	Progressive dyspnoea, non-productive cough and chills	After third dose	Patchy, nodular ground-glass opacities along a bronchovascular distribution throughout the lungs	-Bronchoscopy with BAL was performed and ruled out infectious aetiology. - Many WBCs (576) with lymphocytic predominance.	-Single small fragments of alveolate pulmonary parenchyma showing no histopathologic abnormality - There was no evidence of inflammation, alveolar thickinig or filling, no malignant infiltrates, no granuloma or vasculities	No signs of DAH or confirmed OP	Not mentioned
2\ Alkhayat *et al*.^11^	24\female	Acute onset dyspnoea, non-productive cough, wheezing and rhinorrhea	After first dose	Scattered ground-glass opacities on both lungs	Not done	Not done	No signs of DAH or confirmed OP	Required O2
3\ Katta *et al*.^12^	23\male	Cough, pleuritic chest pain, high grade fever, rash	Not mentioned	Diffuse bilateral ground-glass opacities	-BAL was done and ruled out infectious aetiology - BAL showed lymphocytic alveolitis	Not done	No signs of DAH or confirmed OP	Required O2
4\ Syeda *et al*.^13^	70\male	Progressive dyspnoea, fever, productive cough	Not mentioned	New alveolar opacities involving the lower lobes	Not done	Not done	No signs of DAH or confirmed OP	Required O2
5\ Etori *et al*.^14^	82\female	Dyspnoea on exertion	After third dose	Bilateral diffuse ground-glass opacities	BAL was done and showed lymphocytosis	Biopsy was done and showed interstitial fibrosis, lymphocytic infiltration	No signs of DAH or confirmed OP	Not mentioned
Our case	Female in her forties	Productive cough with expectoration of blood-tinged sputum and increasing SOB	After third dose	Bilateral airspace consalidations, ground-glass opacities, reversed halo sign (atoll sign)	BAL was done and ruled out infectious aetiology -BAL: 250 nucleated cells with macrophage predominance (69%) and significant lymphocytosis (26%)	- Histopathology: no infection, neoplasm, granulomas or signs of pulmonary lymphoma - diffuse alveolar damage and OP	OP was confirmed with no signs of DAH	Required O2 and ICU admission
(B)								
Number\ authors	Pneumonitis grade	Treatment offered	Outcome	Long-term recommendations				
1\ Sabat *et al*.^10^	Grade III	- Empiric antibiotics: Trimethoprim\silfamethaxazole 400 mg q6hr. - Prednisone 80 mg daily - Discontinuation of brentuximab	- Rapid complete resolution of symptoms - follow-up CT scan 4 mo later showed complete resolution of bilateral pulmonary opacities previously seen	- The offending agent should never be prescribed. An alternative medication is recommended - Prednisone taper - Continue total 14 d of levofloxacin 750 mg daily				
2\ Alkhayat *et al*.^11^	Grade III	- Discontinuation of the drug - High-dose glucocorticoids	-Marked improvement with resolution of O2 requirements within one week - Marked improvement in the CT findings one month later	The offending agent should never be prescribed. An alternative medication is recommended				
3\ Katta *et al*.^12^	Grade III	IV steroids	Rapid clinical improvement	Discharged on empiric antibiotics and oral steroid taper				
4\ Syeda *et al*.^13^	Grade III	- Discontinuation of drug - Broad-spectrum antibiotics - IV steroids	- Clinical improvement - CT one month later showed complete resolution bilateral alveolar opacities	-The offending agent should never be prescribed. An alternative medication is recommended - Taper steroid				
5\ Etori *et al*.^14^	Grade III	Discontiuation of the drug	Full recovery	The offending agent should never be prescribed. An alternative medication is recommended				
Our case	Grade IV	- Empiric antibiotics (Ceftriaxone and Azithromycin) - IV methylprednisolone 1 mg\kg twice daily switched into oral prednisone 60 mg daily tapered over 6 weeks	- Full recovery and functional capacity improved - Follow-up CT scan 6 wk later showed complete radiological resolution and lung parenchyma appeared normal	- The offending agent should never be prescribed. An alternative medication is recommended - Steroid taper - Prophylactic Trimethoprim\Sulfamethoxazole on discharge				

BAL, bronchoalveolar lavage; CT, computed tomography; DAH, diffuse alveolar haemorrhage; OP, organizing pneumonia; SOB, shortness of breath; WBC, white blood cell.

* Original work.

When comparing our case to the existing literature, we observed several relevant similarities. The presenting features were productive cough after the third dose of BV, which aligns with the reported cases. Within the documented cases, BV-induced pneumonitis shows a diverse and unpredictable pattern of toxicity, with symptoms appearing at different times among reported cases, indicating a likely idiosyncratic reaction^10–14^. In the reported cases, cough was a prevalent presentation of BV-induced lung injury^10–13^. Most cases had a dry cough, although a productive cough was reported at least once^13^. Additionally, our case’s CT findings showed similarities to the reported cases^10–14^. Our patient displayed bilateral upper lobe ground-glass opacities, with a suggested reversed halo sign, consistent with what’s seen in all mentioned cases^10–14^. However, the anatomical distribution was not consistent suggesting no zonal predominance. In four out of the five cases, the opacities were diffuse and scattered^10–12,14^, while another case reported lower lobe predominance^13^. In contrast, our case exhibited upper lobe predominance with the presence of a reversed halo sign. The reversed halo sign, also known as the atoll sign, is common in cases of OP but has been reported in association with a wide range of pulmonary diseases, including invasive pulmonary fungal infections, tuberculosis, community-acquired pneumonia, and GPA^4^. Furthermore, the observation of DAD with interseptal thickening in our case raised suspicion of BV-induced lung injury.

BAL serves as a crucial tool not only to rule out infections and other potential causes of acute hypoxic respiratory failure but also to aid in diagnosing disorders like DAH. Additionally, BAL can provide valuable pathological findings that may influence the treatment approach. For rare diseases, such as drug-induced pneumonitis, the cell differential obtained from BAL can be particularly helpful; the presence of differential eosinophilia or differential lymphocytosis (>20% lymphocytes as seen in our case) can suggest drug-induced pneumonitis, in contrast to predominant neutrophilia typically observed in infections or neutrophilic granulomatous necrosis seen in GPA vasculitis. In addition to BAL, we conducted a TBBX (transbronchial biopsy) in our case, which definitively confirmed the presence of OP, a pathological finding that was never reported before^15,16^.

Our case strongly contributes by elucidating a rare, life-threatening side effect of BV, manifested as severe acute lung injury, which may advance to severe respiratory failure. Furthermore, it heightens clinical awareness among healthcare professionals regarding this uncommon complication. Early diagnosis and proactive management, involving the discontinuation of the drug and the administration of steroids with or without steroid-sparing agent, play a pivotal role in averting serious consequences and enhancing the overall quality of healthcare services.

The primary constrains of our case report stem from the rarity of the diagnosis, which was only confirmed following the exclusion of all possible causes through the extensive clinical, laboratory, and radiological evaluations. Additionally, the lack of well-established approaches for both the diagnosis and management of our case is a significant limitation, attributable to the infrequency of the diagnosis and the scarcity of the similar case reports.

## Conclusions

BV-induced lung toxicity is a rare yet potentially serious adverse event associated with BV treatment. Healthcare providers should be aware of this rare complication and maintain a high index of suspicion to ensure early recognition and appropriate management. Diagnosing BV-induced lung toxicity typically involves a comprehensive evaluation of clinical symptoms and investigative findings. A Combination of high-resolution CT scan and BAL findings play a crucial role in ruling out infections and other potential differential diagnoses, while also aiding in confirming the disease through findings such as differential lymphocytosis and occasional eosinophilia. Our paper provides a stepwise approach for managing BV-induced lung toxicity. Based on the grade of lung toxicity (I–IV) management may involve temporary or permanent discontinuation of the offending agent. Glucocorticoids and steroid-sparing agents could be used in more advanced stages.

## Ethical approval

The study is exempt from ethical approval in our institution.

## Consent

Written informed consent was obtained from the patient for publication and any accompanying images. A copy of the written consent is available for review by the Editor-in-Chief of this journal on request.

## Source of funding

This research received no external funding.

## Author contribution

O.R.S.K., S.M.A.M., F.O., H.S., H.M., J.A.: study concept or design. O.R.S.K., S.M.A.M., F.O., H.S., J.A.: writing the manuscript. O.R.S.K., F.O., J.A.: review and editing the manuscript. O.R.S.K., J.A., H.M.: data collection. J.A.: supervision. J.A.: treating physician.

## Conflicts of interest disclosure

None.

## Research registration unique identifying number (UIN)

Not applicable.

## Guarantor

Omar R. S. Khalil.

## Data availability statement

Data supporting the study results can be provided, followed by a request sent to the corresponding author’s e-mail.

## Provenance and peer review

Not commissioned, externally peer-reviewed.
